# The association between social capital indicators and psychological distress in Catalan adolescents

**DOI:** 10.3389/fpsyg.2022.964689

**Published:** 2022-08-17

**Authors:** Elena Carrillo-Alvarez, Ana Andrés, Jordi Riera-Romaní, Dario Novak, Míriam Rodriguez-Monforte, Lluís Costa-Tutusaus, Myriam Guerra-Balic

**Affiliations:** ^1^Global Research on Wellbeing (GRoW) Research Group, Blanquerna School of Health Sciences, Ramon Llull University, Barcelona, Spain; ^2^Faculty of Psychology, Education and Sport Sciences Blanquerna, Ramon Llull University, Barcelona, Spain; ^3^Research Group on Pedagogy, Society and Innovation (PSITIC), Faculty of Psychology, Education and Sport Sciences Blanquerna, Ramon Llull University, Barcelona, Spain; ^4^Faculty of Kinesiology, University of Zagreb, Zagreb, Croatia; ^5^Research Group on Health, Physical Activity and Sport (SAFE), Faculty of Psychology, Education and Sport Sciences Blanquerna, Ramon Llull University, Barcelona, Spain

**Keywords:** social capital, mental health, adolescents, psychological distress, ecological approach

## Abstract

According to the WHO, globally, one in seven adolescents experiences a mental disorder, being in a detrimental situation toward educational achievement, social cohesion, future health and life chances. Calls to identify risk and resilience factors to develop effective preventive actions have been made. Following a systemic approach, we conducted a cross-sectional study on the relationship between social capital and psychological distress in a sample of Catalan adolescents in Barcelona, taking into account a range of other relevant aspects at different levels influencing mental health, including gender, age, migrant status, family background, lifestyle factors, body mass index, and self-rated health. Data were collected through validated questionnaires in December 2016 from 646 of 14- to 18-year-old adolescents from three public and private high schools in Barcelona (Spain). Data analysis included descriptive analysis, a correlational study and logistic regression to obtain the odds ratio for social capital indicators to be associated with psychological distress. Our results suggest that reporting higher levels of family support and higher levels of teacher-student trust reduce the likelihood of suffering psychological distress. Higher levels of neighborhood informal control were associated with mental health, but a possible detrimental effect cannot be ruled out. Being a girl, reporting low self-rated health or higher media use was also associated with higher likelihood of psychological distress. Current results may encourage interventions that focus on social capital as a means to reduce psychological distress and foster well-being in youth.

## Introduction

According to the WHO, globally, one in seven adolescents experiences a mental disorder. Mental disorders account for 13% of the global burden of disease for adolescents and mood disorders are among the leading causes of disability (World Health Organization, 2022). Data from Spain echoes the global trends. The Spanish National Health Survey indicates that the prevalence of mental health and behavioral issues among children in 2017 was of 1 and 3% respectively, with these figures reaching 4 and 6.9% in 2021 (UNICEF, 2020; Perazzo Aragoneses et al., 2021). The COVID pandemic has taken an additional toll on youth mental health (Panchal et al., 2021; Racine et al., 2021). However a point has been made that the pandemic may have not created a new burden of disease, but actually exacerbated preexisting trends in the adolescent population (Hafstad and Augusti, 2021). Research shows that not all youth face the same risks. Adolescents in vulnerable contexts are more prone to develop mental health issues (World Health Organization, 2022), being put in a detrimental situation toward educational achievement, social cohesion, future health and life chances (Mathieson and Koller, 2006; Morgan, 2011; Gómez-López et al., 2019; World Health Organization, 2022).

Calls have been made to identify vulnerable groups and to develop effective preventive actions (Viner et al., 2012; Elgar and Currie, 2016; Panchal et al., 2021; Robinson et al., 2021; Thorisdottir et al., 2021). In this sense, Currie and Morgan (2020), drawing on Bronfenbrenner’s model of human development and data from the international Health Behavior in School-Aged Children (HBSC) study 1983–2020, conducted a scoping review to frame the evidence on the determinants of adolescent mental health across system levels.

At the Individual level, which has been the most researched one, sex/gender, migrant status, age, sexual orientation, adoption, and farm-living were identified as relevant for adolescent mental health. In general, boys reported better results than girls in several mental health indicators (Levin et al., 2009; Savoye et al., 2015). With regards to the migrant status, adolescents who themselves or their parents are not native, experience worse mental health. However, more detailed analysis seems to indicate that once differences in gender, age, family affluence and school environment are taken into account, differences due to immigrant status fade (Molcho et al., 2010; Walsh et al., 2010). HBSC data for age is scarce, although a tendency that older adolescents are more likely to experience mental health issues can be identified which is consistent with other empirical evidence (Costa-Tutusaus and Guerra-Balic, 2016; Bullón et al., 2019) and with the general observation of adolescence being the stage of development where the majority of mental disorders emerge (De Girolamo et al., 2012).

Lifestyle factors and health-related behaviors have also been analyzed across various studies using HBSC data. Some of the results suggest that overweight is a strong predictor of psychosomatic complaints (Whitehead et al., 2017) and the correlation of obesity and high social media use with a lower self-rated life satisfaction and quality of life (Bullón et al., 2019; Baile et al., 2020). Sleep duration and difficulties to sleep have also been correlated with worse mental health (Vandendriessche et al., 2019). Inversely, physical activity and following a healthy dietary pattern, have been associated with better life satisfaction and self-esteem and better general mental health, respectively (Jacka et al., 2011; O’Neil et al., 2014; Khalid et al., 2016; Meyer et al., 2021). Some of the results suggest that overweight is a strong predictor of psychosomatic complaints (Whitehead et al., 2017), and a correlation between obesity and high social media use with a lower self-rated life satisfaction and quality of life (Bullón et al., 2019; Baile et al., 2020). Sleep duration and difficulties to sleep have also been correlated with worse mental Health (Vandendriessche et al., 2019). Inversely, physical activity and following a healthy dietary pattern, have been associated with better life satisfaction and self-esteem and better general mental health, respectively (Jacka et al., 2011; O’Neil et al., 2014; Khalid et al., 2016; Meyer et al., 2021).

Data from the HSBC study show that, at the Microsystem level, family and friends’ support and communication, family socioeconomic status, and the peer and school’s social environments are important predictors of adolescent mental health (Currie et al., 2004; Moreno et al., 2009; Freeman et al., 2012; Matos et al., 2014; Nielsen et al., 2015; Calmeiro et al., 2018; Moore et al., 2018; Inchley et al., 2020). Social capital in the community domain was also found to moderate the association between socioeconomic status and life satisfaction, internalizing and psychosomatic symptoms, and overall emotional wellbeing (Buijs et al., 2016). At the Macro level, inequality has been described as a driver of mental health disturbances for adolescents and adults (Currie et al., 2008; Pickett and Wilkinson, 2010; Currie and Morgan, 2020).

Overall, these data show how mental health in adolescents is the result of a complex intricacy of multiple factors. The more risk factors adolescents are exposed to, the greater the potential impact on their mental health. On the contrary, resilience factors can contribute to alleviate the effect of the negative ones. The aim of this study is to investigate social capital as an asset for adolescent mental health in sample high-school students in Barcelona (Spain). To do so, we mimic Novak and Kawachi’s study on social capital and mental health in Croatian adolescents.

Several definitions of social capital exist (Lochner et al., 1999; Paldam, 2000), which despite different nuances share the view that social capital can be an asset for societies in that it recognizes the importance of informal and formal networks in supporting individuals and communities through difficult times and increases their opportunities for improving and sustaining health through better access to information and resources (Ottejberg, 2005). A lifecourse perspective to social capital has been brought out to recognize that the social space and experience of children and adolescents differs from that of adults. For example, it has been posited that bonding social capital could be more important in the early stages of life, and that, especially in this period, social capital and social networks extend beyond the geographical limits (McPherson et al., 2013). In children and youth, social capital is typically assessed through a set of indicators that draw on Virginia Morrow’s qualitative work on youth social capital (Morrow, 1999), and include sense of belonging (identity and safety with local environments); autonomy and control (perceptions of power to influence community/institutional decisions); and social networking (participation in school and community life). Social capital is typically assessed either at the adolescent and parents’ level.

Social capital has been linked to a variety of outcomes in young people, ranging from risk behaviors, overweight, dietary habits, and physical activity (Singh et al., 2008; Duke et al., 2012; Singh and Ghandour, 2012; Nesbit et al., 2014; Novak et al., 2015, 2017). The study of social capital and health in adolescents do not only focus on physical aspects of health, but also on psychological ones (Yi et al., 2009; Han, 2012; Novak and Kawachi, 2015; Mohammadi et al., 2022). McPherson et al. (2013) undertook a review of studies to explore the relation of social capital and adolescent mental health and concluded that such relation existed for family and community-based social capital. To our knowledge, no data exist on Spanish populations.

Based on the former, we depart from the work by Novak and Kawachi (2015) and from the HSBC evidence collected by Currie and Morgan (2020) to conduct a cross-sectional study on the relationship between social capital and psychological distress in a sample of Catalan adolescents in Barcelona. With that aim, we consider a range of other relevant aspects at different system levels influencing mental health, including gender, age, migrant status, parental migrant status, family affluence, parental educational level, lifestyle factors (nutrition, physical activity, toxic habits, technological leisure, hygiene), body mass index (BMI), and self-rated health (SRH). In this way, we aim to identify risk and protective factors for psychological distress among Catalan adolescents.

Based on the literature reviewed, we hypothesize: (1) Lower family affluence, lower parental education, immigrant status of the adolescent and/or the family and female gender will be associated with higher levels of psychological distress; (2) Better lifestyle scores will correlate with lower levels of psychological distress; (3) Higher social capital scores will be associated with lower levels of psychological distress; (4) BMI higher than 25 kg/M2 will be associated with higher levels of psychological distress; (5) Good, very good or excellent SRH will be associated with lower levels of psychological distress.

## Materials and methods

### Design and participants

A descriptive cross-sectional study was carried out in a sample of 646 adolescents aged 14- to 18 years old from three public and private high schools in Barcelona (Spain). For the sample recruitment, all public high schools in Barcelona (*n* = 71) were emailed an invitation letter to participate in the study; also, several private schools (*n* = 5) were contacted through personal contacts of the research team. Finally, one public high-school and two private schools agreed to participate in the study.

### Data collection and study variables

Data collection was performed in December 2016, in each high school, in a scheduled appointment by one or two members of the research team, who were always present during adolescents’ response, providing indications and solving doubts when needed. Participants were handed a folder that contained all data collection instruments and were allocated 40 min to answer them. Anonymity was preserved by not collecting any personally-identifying information.

The study had been approved by the Ethical Board Committee of Blanquerna School of Health Sciences and all eligible students and their parents were informed about the aim, procedure and data collection tools. Except eight eligible participants, all other returned the informed consent form signed by their parents and by themselves, therefore entering the study.

The participants responded to sociodemographic questions, which included gender (male or female), age, country of birth and parental country of birth, level of education for both parents and family wealth. Age was recorded by asking the participants’ birth month and year. Country of birth was grouped into autochthonous and otherwise, as it was parental country of birth (both parents autochthonous and otherwise). For each parent, participants provided the highest level of education achieved (no studies, primary school, compulsory secondary school, non-compulsory secondary school, vocational training, university studies) and responses were dichotomized considering vocational training, university studies as higher educational level.

Family wealth was assessed through the Family Affluence Scale (FAS III). The FAS III scale is the instrument used in the World Health Organization’s Health Behavior in School-aged Children (HBSC) study to assess socioeconomic position and provides a 0–9 score that is typically categorized into low affluence (0–2), middle affluence (3–5) and high affluence (6–9) for categorical analysis (Boyce et al., 2006). While it is reliability in high-income countries has been questioned due to the improvement of general living conditions in the last decades, recent analysis show that it is a valid measure to identify low-income families in such settings (Corell et al., 2021). In our analysis, responses 3–9 were grouped to dichotomize the variable into low family affluence (responses 0–2) and high family affluence (responses 3–9).

Outcome variable psychological distress was assessed through the KESSLER k-6 scale (Kessler et al., 2010). Previous studies have shown that the K6 is a reliable instrument able to detect mood and anxiety in adolescents (Green et al., 2010). The six items assess the frequency in which respondents felt nervous, hopeless, restless/fidgety, depressed that nothing could cheer them up, everything was an effort, and worthless. Each question is scored from 0 (None of the time) to 4 (All of the time). Scores of the 6 questions were then summed (0–24) with scores of 13 or higher indicating high levels of psychological distress.

Social capital in the family, school and neighborhood setting was measured with a 6-item multi-setting adapted scale on family, school and neighborhood social capital adapted from Furuta et al. (2012). Family support in school was assessed by the single item: “Do you feel your family understands and gives attention to you during high school?.” Neighborhood social capital was assessed by two items: “Do you feel people trust each other in your neighbourhood (neighbourhood trust)?”; “Do you feel that your neighbours step in to criticize deviant behavior among high school students (informal social control)?.” School social capital was assessed by three questions; “Do you feel teachers and students trust each other in your high school (teacher-student interpersonal trust)?”; “Do you feel students trust each other in your high school (student interpersonal trust)?”; “Do you feel students collaborate with each another in your high school (students’ collaboration in school)?.” The response options to all questions were on a 6-points Likert scale where 1 was “strongly disagree” and 6 “strongly agree.” Responses 1–3 and 4–6 were combined to create a dichotomous variable indicating low and high perceived social capital, respectively (Novak and Kawachi, 2015).

The VISA-TEEN questionnaire (Costa-Tutusaus and Guerra-Balic, 2016) was used to evaluate the lifestyle dimensions. This questionnaire evaluates overall lifestyle, as well as its sub-dimension nutrition [compliance with the Catalan 2012 Food-based dietary guidelines, plus water and other beverages’ intake], physical activity [weekly moderate and high intensity practice], use of technologic leisure [hours dedicated to social networks and electronic games, sleep hours], toxic habits [tobacco, alcohol and other drugs’ consumption] and hygiene [hands and teeth washing]. The total mark is 45, and each dimension’s score ranges from 0 to 3, with higher values indicating more alignment with health recommendations.

Self-rated health (SRH) was assessed using the standard single item question: “How do you perceive your health?” (Ware and Sherbourne, 1992). Possible responses ranged from 1 (very poor) to 5 (excellent). Responses were dichotomized being scores 3–5 considered as “good SRH” and responses 1–2 “poor SRH” health. Body mass index (BMI) was used based on self-reported height and weight. Was stablished as a cut-off to discriminate respondents with and without overweight/obesity, based on the recommendations of the Spanish Association for the Study of Obesity (SEEDO, 2016).

### Data analysis

Data analysis was performed with SPSS v21 (IBM, 2019) and included descriptive analysis, a correlational study and logistic regression to obtain the odds ratio for social capital indicators to be associated to psychological distress.

The first steps of the data analysis entailed the descriptive and correlational analyses. The preliminary tasks to these procedures correspond to the verification of the normal distribution for the dependent variables using the Kolmogorov–Smirnov test (none of our continuous variables showed a normal distribution); verification of variance homogeneity; and dichotomization of the dependent and predictor variables (except for age and lifestyle variables for which a cut-off score is not available and were therefore left in continuous mode). In all cases, 1 represented the values that are hypothesized to be associated with higher psychological distress (value 1 in the dichotomized K6 score as well).

In our study, all continuous variables were non-normal. Therefore, correlational analysis was conducted using Spearman Rank Correlation, which is an appropriate test when one or both variables are ordinal and/or normality assumptions are not met. We also compared the means for the dependent and social capital variables based on gender, parental level of studies, family affluence, and migrant status of both the adolescent and their parents in their dichotomized form using t-student test.

Using odds ratio to present the results is recommended when the study is interested in the impact of the independent variables, controlling for the effect of the others, as well as to prevent the full effect of the true impact of a unit change in independent variables on the outcome variable (Morgan and Teachman, 1988; DeMaris, 1995). The purpose of this study was to assess the significance of social capital variables in contributing to the dependent variable, psychological distress, controlling for the other analysis variables: gender, age, parental level of studies, family affluence, migrant status of the adolescent and their parents, BMI, SRH and lifestyle indicators. To include variables in the logistic regression model we used forward stepwise selection. Predicted probabilities of an event occurring were determined by Exp (B). For all study analysis, we consider a significance value of 0.05.

## Results

### Characteristics of the sample

The sample consisted of a total of 654 students, 345 male (52.7%) and 309 female (47.2%). 90.1% of the students were born in Spain. 33.38 were enrolled in compulsory secondary school, 47.25% in post-compulsory secondary school and 19.35% in different degrees of vocational training. Regarding parental education, university studies predominate (42.4% in the case of mothers, 35.7% in the case of fathers). 82.6% of mothers and 81.1% of fathers were native from Spain. Both parents were born in Spain for 78.7% of the sample. 70.1% of the sample was classified as having high family affluence, 25.3% as middle-income and 2.3% as low.

In terms of covariates, mean lifestyle scores ranged between 2.00 (*SD* = 0.50) for the dimension of nutrition and 2.48 (*SD* = 0.79) for the dimension of physical activity, except for technological leisure that obtained the lowest score with a 1.61 (*SD* = 0.67). 10% of the sample was overweight, and over 16% reported poor or very poor self-rated health. For social capital scores, the highest mean score was for family support (*M* = 4.3; *SD* 1.41), followed by student trust (*M* = 3.85; *SD* 1.31); teacher-student trust (*M* = 3.59; *SD* 1.33); neighborhood informal control (*M* = 3.08; *SD* 1.43); neighborhood trust (*M* = 2.97; *SD*1.41) and collaboration among students (*M* = 1.78; *SD* 1.25). Complete data on the sample characteristics are shown in Table 1.

**Table 1:** Descriptive statistics of the sample.

Variable	Category	N (%)	Variable	Category	N (%)
Gender	Male	345 (52.7)	Maternal level of studies	None	10 (1.5)
	Female	309 (47.1)		Primary education	23 (3.5)
Course	Compulsory Secondary school	219 (33.38)		Compulsory secondary education	86 (13.1)
	Post-compulsory secondary school	310 (47.25)		Post-compulsory secondary education	68 (10.4)
	Vocational Training	127 (19.35)		Vocational training	176 (26.8)
SRH	Poor or very poor	106 (16.2)		University studies	278 (42.4)
	Fair. good or very good	540 (82.3)	Paternal level of studies	None	16 (2.5)
Family wealth	Low	15 (2.3)		Primary education	28 (4.3)
	Middle	166 (25.3)		Compulsory secondary education	92 (14.0)
	High	460 (70.1)		Post-compulsory secondary education	62 (9.5)
BMI	<25	547 (83.4)		Vocational training	206 (31.4)
	> = 25	66 (10.1)		University studies	234 (35.7)
Psychological distress	Yes	156 (23.8)			
	No	476 (72.6)			
**Variable**		**X (SD)**	**Variable**		**X (SD)**
Age		15.72 (1.23)	Family support		4.3 (1.41)
BMI		21.23 (3.31)	Neighborhood trust		2.97 (1.41)
Nutrition		2.00 (0.50)	Neighborhood informal control		3.08 (1.43)
Physical activity		2.48 (0.79)	Teacher-student trust		3.59 (1.33)
Hygiene		2.36 (0.70)	Student trust		3.85 (1.29)
Toxic habits		2.52 (0.66)	Collaboration among students		1.78 (1.25)
Technological leisure		1.61 (0.67)			
Lifestyle		33.29 (4.84)			

Mean comparisons showed significant differences for K6 scores for family affluence, parental migrant status, and gender (Table 2). All social capital indicators showed significantly different scores based on the FAS scale except for neighborhood informal control. Native-born students obtained significantly higher scores on student-teacher trust, and those with families born outside Spain had lower scores on trust among students. Girls obtained lower scores in all SC indicators, but only differences in family support, neighborhood trust, teacher-student trust and trust among students were significant. Students whose mother’s had higher education (vocational training or university students) had significantly higher scores all social capital indicators except for neighborhood informal control. In the case of paternal education, differences for social capital indicators were only significant for neighborhood trust and student trust. Differences in psychological distress based on parental education were not significant.

**Table 2:** Mean differences for psychological distress and social capital scores based on gender, adolescent and parental migrant status, family wealth and parental educational level.

	Gender		Adolescent migrant status		Parental migrant status		Family wealth		Mother’s educational level		Father’s educational level	
	Girl (*n* = 309)	Boy (*n* = 346)	Migrant (*n* = 64)	Native (*n* = 591)	Migrant (*n* = 138)	Both Native (*n* = 516)	Low income (*n* = 460)	Middle-high income (*n* = 181)	Low (*n* = 187)	High (*n* = 454)	Low (*n* = 196)	High (*n* = 440)
Family support	4.16^*^	4.43^*^	4.02	4.33	3.85	4.31	3.97^***^	4.43^***^	4.24^*^	4.34^*^	4.25	2.36
Neighborhood trust	2.85^*^	3.08^*^	2.95	2.98	2.69	2.82	2.69^**^	3.08^**^	2.83^*^	3.04^*^	2.76^*^	3.06^*^
Neighborhood informal control	3.00	3.16	3.15	3.15	3.20	2.97	2.93	3.13	2.94	3.14	2.92	3.13
Teacher-student trust	3.47^*^	3.69^*^	3.87^*^	3.56^*^	3.63	3.82	3.41^*^	3.64^*^	3.38^*^	3.66^*^	3.48	3.65
Student trust	3.73^*^	3.96^*^	3.97	3.84	3.69^*^	4.18^*^	3.65^*^	3.92^*^	3.60^**^	3.96^**^	3.71^*^	3.93^*^
Collaboration among students	3.73	3.82	3.89	3.77	3.66	4.05	3.57^*^	3.86^*^	3.59^*^	3.85^*^	3.72	3.76
Psychological distress	10.54^***^	8.63^***^	10.07	9.48	10.94^**^	8.52^**^	9.14^***^	10.55^***^	9.88	9.35	9.79	9.30

* *p* ≤ 0.5;

** *p* ≤ 0.01;

*** *p* ≤ 0.001.

### Relationship between social capital and psychological distress

All social capital indicators significantly correlated with psychological distress in an inverse way (Table 3). The strongest association was for family support, and the lowest for neighborhood informal control. Psychological distress was also significantly associated with FAS, SRH and technological leisure. In terms of social capital indicators, family support was associated with FAS, SRH, BMI, technological leisure, and toxic habits; neighborhood trust with FAS, nutrition and technological leisure, neighborhood informal control with technological leisure, teacher-student trust with FAS, SRH, BMI, age and technological leisure, student trust was associated with age, and collaboration among students with age and physical activity.

**Table 3:** Results of the correlational analysis.

	Family support	Neighborhood trust	Neighborhood informal control	Teacher-student trust	Student trust	Collaboration among students	Psychological distress
Psychological distress	−0.311^***^	−0.204^***^	−0.113^**^	−0.207^***^	−0.121^**^	−0.122^**^	
Family wealth	0.152^***^	0.122^**^	0.061	0.102^*^	0.110^**^	0.108^*^	−0.076^*^
Self-rated health	−0.193^***^	−0.068	−0.068	−0.105^*^	−0.052	−0.058	0.198^**^
Body Mass Index	−0.086^*^	−0.045	−0.031	−0.117^**^	−0.029	−0.014	0.032
Age	0.066	−0.022	0.047	0.092^*^	0.122^**^	0.133^***^	−0.029
Nutrition	0.053	0.082^*^	0.057	−0.028	0.000	0.037	0.069
Physical Activity	0.017	0.073	0.034	0.035	−0.016	−0.104^*^	−0.046
Technological leisure	0.190^***^	0.099^*^	0.080^*^	0.104^*^	0.022	−0.019	−0.162^***^
Toxic Habits	0.114^**^	0.013	−0.065	0.020	−0.042	−0.020	−0.073
Hygiene	0.076	0.059	0.027	0.062	−0.008	0.044	0.003

* *p* ≤ 0.5;

** *p* ≤ 0.01;

*** *p* ≤ 0.001.

The association between social capital and psychological distress when considering the rest of covariates is shown in Table 4. In the overall model, high psychological distress was significantly inversely associated with three domains of social capital: family support (OR 3.483; 95% CI 2.035–5.961), neighborhood informal control (OR 1.809; 95% CI 1.025–3.192), and teacher-student trust (OR 1.950; 95% CI 1.163–3.272). In our sample, gender and the educational level of the father remained significantly associated with psychological distress. Girls were 3.198 times more likely to experience psychological distress (95% CI 1.887–5.420) and those adolescents whose father had not achieved higher education were 1.735 times more likely to experience psychological distress (95% CI 1.036–2.905).

**Table 4:** Odds ratios for high psychological distress in the sample.

		Family SC	Neighborhood SC	School SC	All domains SC
		Model 1	Model 2	Model 3	Model 4
		OR(CI)	OR(CI)	OR(CI)	OR(CI)
Family support	Low	4.044 (2.393–6.835)^***^			3.483 (2.035–5.961)^***^
	High				
Neighborhood trust	Low				
	High				
Neighborhood informal control	Low		2.411 (1.401–4.150)^**^		1.809 (1.025–3.192)^*^
	High				
Teacher-student trust	Low			2.374 (1.449–3.889)^*^	1.950 (1.163–3-272)^*^
	High				
Student trust	Low				
	High				
Collaboration among students	Low				
	High				
Gender	Girl	3.079 (1.832–5.174)^***^	3.008 (1.814–4.990)^***^	2.797 (1.682–4.652)^***^	3.198(1.887–5.420)^***^
	Boy				
Self-rated health	Low	0.496 (0.270–0.910)^*^	0.371 (0.208–0.662)^**^	0.430 (0.239–0.771)^**^	
	High				
Paternal level of studies	Low				1.735 (1.036–2.905)^*^
	High				
Technological leisure				0.687 (0.473–0.999)^*^	

* *p* ≤ 0.5;

** *p* ≤ 0.01;

*** *p* ≤ 0.00.

*OR, odds ratio; CI, confidence interval*.

Model 1. Association between gender, age, migrant status, parental migrant status, family affluence, parental educational level, lifestyle factors, BMI, SRH and family social capital.

Model 2. Association between gender, age, migrant status, parental migrant status, family affluence, parental educational level, lifestyle factors, BMI, SRH and neighborhood social capital.

Model 3. Association between gender, age, migrant status, parental migrant status, family affluence, parental educational level, lifestyle factors, BMI, SRH and school social capital.

Model 4. Association between gender, age, migrant status, parental migrant status, family affluence, parental educational level, lifestyle factors, BMI, SRH and all social capital variables.

## Discussion

This is, to our knowledge, the first study to investigate the relationship between social capital and psychological distress in a sample of Catalan adolescents. Following the socio-ecological model of Bronfenbrenner, a range of other relevant aspects for mental health have been considered. Bronfenbrenner’s model understands childhood development as the result of a complex system of relationships affected by multiple levels including individual features and the surrounding layered environments, from immediate settings of family and school to broad cultural values, laws, and customs (Bronfenbrenner and Morris, 1998). In our study, gender, age, migrant status, parental migrant status, family affluence, parental educational level, lifestyle factors (nutrition, physical activity, toxic habits, technological leisure, hygiene), BMI, and SRH were considered when looking at the relation between aspects of social capital and psychological distress.

Our results suggest that the likelihood of suffering psychological distress is lower when reporting higher levels of family support or higher levels of teacher-student trust. In the complete model, higher levels of neighborhood informal control were also associated with our mental health indicator, but based on the CI range, adverse effects could not be ruled out (OR = 1.765, 95% CI: 0.998–3.121). These results are aligned with previous research observing that family relations offer a prominent protective role for youth’s mental health (Morgan and Haglund, 2009; Rothon et al., 2012; McPherson et al., 2013; Lindfors et al., 2017; Hirota et al., 2021).

In our study, family support was the strongest predictor of lower levels of psychological distress, with those reporting lower levels of family support having 3, 6 times more chances of mental hazards. Attempts to elucidate the mechanisms beneath this link include positive communication, norms shaping, the fact that time together prevents from other activities, an increased sense of belonging and a more secure attachment, and that closer relations with the parents allow them to identify signs of alarm. Indeed, families provide the ground for a healthy psychosocial functioning, including emotion processing, social competence, health beliefs and management, and adjustment of the stress-responsive biological regulatory systems, which are critical for mental health (Repetti et al., 2002; Zdanowicz et al., 2004, 2011; Kent and Bradshaw, 2021). The study of family social capital in relation to children’s wellbeing goes back to Coleman (1988, p: S111). However, more research on family social capital has been deemed necessary to better understand the role of social influences on overall and mental health (Rothon et al., 2012; Carrillo et al., 2017).

The result of teacher student-trust being significantly associated with lower odds of reporting psychological distress is consistent with another research on the field. Lindfors et al. (2017) found out that school social capital conceptualized as the positive and supportive relationships between students and teachers predicted lower school burnout in Finish high-school students. School bonding was also negatively associated with severe mood and behavior disorders in US adolescents (Hirota et al., 2021). The school environment has been found to be critical for students to develop confidence, a sense of efficacy, and a positive sense of community (Te Wang et al., 2013; Krane et al., 2016; Aldridge and McChesney, 2018). Explanations for these associations rely on psychological theories such as the attachment theory by Bowlby or the developmental systems theory of Bronfenbrenner (Sabol and Pianta, 2012).

Our study is not the first one to observe a possible detrimental effect of high informal control in the neighborhoods on mental health outcomes (Murayama et al., 2015; Novak et al., 2015; Ferlander et al., 2016). Villalonga-Olives and Kawachi (2017) extensively wrote about the dark side of social capital, something that had been previously addressed by authors like Portes and Landolt (1996) or Campos-Matos et al. (2016). Informal social control refers to the role of members of a community in stepping in to prevent the occurrence of “deviant” behaviors. As such, it can result in excessive demands by the members of tightly knit groups, restrictions on individual freedom, exclusion of out-group members, and the disregard of members’ aspirations (Villalonga-Olives and Kawachi, 2017).

Based on the literature reviewed, we had also hypothesized that lower family affluence, lower parental education, immigrant status of the adolescent and/or the family and female gender would be associated with higher levels of psychological distress. In the present study only gender and paternal level of education remained significant in the final model. These results compare to previous research highlighting the importance of family background in terms of migrant status (Delaruelle et al., 2021) and wealth (Currie et al., 2004; Inchley et al., 2020). Nevertheless, as those authors point out, such associations are tightly interrelated and need to be understood in an ecological manner in such a way that the composition of the sample in terms of level of parental background and family experiences may blur or accentuate these differences.

Using data of the Spanish Health Survey, Sonego et al. (2013) researched the influence of parental education on children and adolescent mental health. Their results showed a strong association between both variables during childhood, which outweighed the effect of other determinants such as income or parental occupation, but this association faded as age increased. The “equalizing” effect of adolescence has been previously described (West, 1997). The authors beg caution at interpreting that adolescence removes the effect of detrimental conditions during childhood arguing that the increase of other contexts’ importance (peers, school, community) may dilute the effect of the parental variables, but not erase it. Substantial research backs the lasting effect of childhood living circumstances (Goodman et al., 2011; Lee et al., 2021). Analysis on a subsample of the Somego et al. study, for which specific data was available, seemed to indicate that mental health outcomes were better for those youth in families where both parents devoted themselves to childcare and the percentage of this type of family increases with parental educational level. This appreciation would reinforce our finding of family social capital as the strongest predictor of mental health in adolescents.

Despite gender information was collected in our study in a binary form, omitting other possible gender identities that may be relevant for mental health (Aparicio-García et al., 2018; Chew et al., 2020; de Graaf et al., 2021), our results indicate that gender greatly influences the odds of suffering ill mental health. The gender gap in mental health has been widely described. Campbell et al. (2021) compared data from 78 countries concluding that girls do worse than boys worldwide despite the sociocultural differences across nations. Possible explanations included the excessive social demands to girls, the existence of sometimes contradictory gender norms (i.e., being femininely attractive, being high achieving, and being “one of the boys”), aesthetic and body image issues and inherent differences in personal characteristics such as self-esteem, stress coping, support seeking and emotional regulation (Bolognini et al., 1996; Smolak, 2004; Eschenbeck et al., 2007; Plaesu, 2011; Piko, 2017).

In terms of lifestyle, we anticipated that better scores in the five evaluated dimensions would correlate with lower levels of psychological distress. However, no dimension remained significantly associated to mental health in the final model. Better SRH was also associated with lower psychological distress. It has been argued that SRH represents an irreplaceable dimension of health status, capturing a myriad of factors that not only represent the subjective experience of a person’s wellbeing, but also is an independent predictor of mortality (Idler and Benyamini, 1997; Waller, 2015). Several studies demonstrate that mental health and SRH are closely linked, and their relation has been studied in both directions (SRH as an influence for mental health, and vice versa; Wade and Vingilis, 1999; Lachytova et al., 2017; Craig et al., 2018). In our case, being both measures subjective and self-reported it is possible that other individual characteristics such as personality, self-concept or self-esteem may influence this association (Vingilis et al., 2002; Arsandaux et al., 2019).

Our results stress the fact that influences on adolescent mental health are multiple and interact to each other in a synergic but not decisive way. Additionally, our results need to account for some limitations. First, the cross-sectional nature of the study does not allow to establish causation nor rule-out reverse causation (i.e., having mental health issues promote lower levels of social capital). Second, we have obtained the data from a convenience sampling strategy that may have hindered those from detrimental contexts to participate in the study. Only around 15–20% of the sample belonged to low-income families, had a migrant background or parents had only compulsory education. Third, all our measures are subjective, therefore the possibility of personal bias in the data exist. Fourth, there is no gold standard in the measurement of social capital. We have used a limited selection of social capital indicators from different domains, and discrepancies with other evidence may be the result of a different operationalization of such social capital indicators. Importantly, we have not explicitly considered the influence of online social capital, which is increasingly being recognized as an important source of social capital, particularly for the younger generations. Last, our analysis does not account for personal or family data on mental health or relevant psychological information that can enhance current results such as family dynamics, personality, or coping styles. However, we offer a systemic view of risk and protective factors for adolescent mental health, with a special focus on social capital. Moreover, we frame our research in a robust framework, which has been identified as necessary in the field (McPherson et al., 2013).

## Conclusion

The recent increase in mental health problems in youth have emphasized the need to identify risk and resilience factors that can contribute to operationalize programs and policies to prevent mental health burdens in this population. Our findings add to the existing body of literature by providing more evidence on the role that social capital can play in the promotion of mental health for adolescents and may encourage interventions. Social capital in the family domain appears to be of particular relevance, with the school and neighborhood environment also playing a role. Social capital is not something tangible that can be acted upon. Rather, actions need to be addressed to components of social capital such as social interaction, interconnectedness, trust or social norms, or to other elements of the system that can condition those (Eriksson, 2011; Ungar, 2011; Villalonga-Olives et al., 2018). Examples of such actions in the family area include interventions directed to promote positive parenting skills or to build parental social capital, like educational and support programs and community dynamization, but also more structural actions like conciliation measures. Actions in the school setting could involve the inclusion in the school educational plan of measures that foster teacher-student relationship, like small group spaces or the implementation of tutorship programs, especially for those in most vulnerable positions.

## Data availability statement

The raw data supporting the conclusions of this article will be made available by the authors, without undue reservation.

## Ethics statement

The studies involving human participants were reviewed and approved by Ethical Board Committee of Blanquerna School of Health Sciences. Written informed consent to participate in this study was provided by the participants, the participants’ legal guardian/next of kin and the school board.

## Author contributions

EC-A, LC-T, MG-B, DN, and JR-R: conception or design of the work. EC-A, LC-T, and MG-B: data collection. EC-A, AA, and LC-T: data analysis and interpretation. EC-A: drafting the article. All authors contributed to the article and approved the submitted version.

## Funding

This project was funded by an APR grant by the Blanquerna School of Health Sciences 2015–16.

## Conflict of interest

The authors declare that the research was conducted in the absence of any commercial or financial relationships that could be construed as a potential conflict of interest.

## Publisher’s note

All claims expressed in this article are solely those of the authors and do not necessarily represent those of their affiliated organizations, or those of the publisher, the editors and the reviewers. Any product that may be evaluated in this article, or claim that may be made by its manufacturer, is not guaranteed or endorsed by the publisher.
